# Thermally cross-linkable fluorene-based hole transporting materials: synthesis, characterization, and application in perovskite solar cells†

**DOI:** 10.1039/d3ra03492e

**Published:** 2023-09-08

**Authors:** Deimante Vaitukaityte, Artiom Magomedov, Kasparas Rakstys, Simon Kwiatkowski, Egidijus Kamarauskas, Vygintas Jankauskas, Jolanta Rousseau, Vytautas Getautis

**Affiliations:** a Department of Organic Chemistry, Kaunas University of Technology Radvilenu pl. 19 Kaunas 50254 Lithuania vytautas.getautis@ktu.lt; b Department of Chemical and Biological Engineering, University of Colorado Boulder CO 80309 USA; c Univ. Artois, CNRS, Centrale Lille, Univ. Lille, UMR 8181–UCCS–Unité de Catalyse et Chimie du Solide, Faculty of Science Jean Perrin Rue Jean Souvraz SP 18 F-62300 Lens France; d Institute of Chemical Physics, Vilnius University Sauletekio al. 3 Vilnius 10257 Lithuania

## Abstract

Perovskite solar cells are among the most promising photovoltaic technologies in academia and have the potential to become commercially available in the near future. However, there are still a few unresolved issues regarding device lifetime and fabrication cost of perovskite solar cells in order to be competitive with existing technologies. Herein, we report small organic molecules with introduced vinyl groups as hole transporting materials, which are capable of undergoing thermal polymerization, forming solvent-resistant 3D networks. Novel compounds have been synthesized from relatively inexpensive starting materials and their purification is less time-consuming when compared to polymers; therefore this type of hole transporter can be a promising alternative to lower the manufacturing cost of perovskite solar cells.

## Abstract 2

Organic semiconductors containing two vinyl groups can undergo thermal cross-linking polymerization at 214 °C leading to higher hole mobilities compared to uncross-linked films and significantly improved efficiencies of perovskite solar cells.

## Graphical abstract

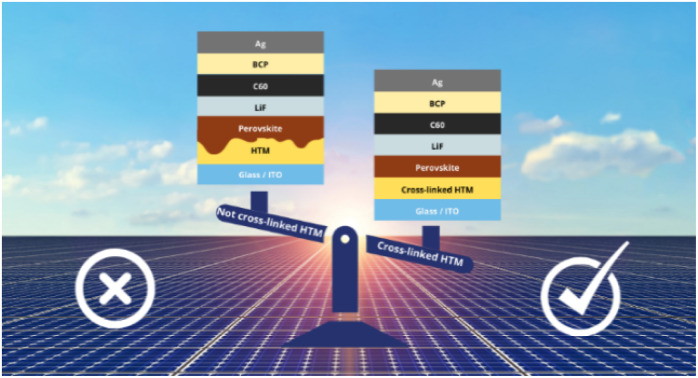

## 1. Introduction

Organic–inorganic hybrid perovskite solar cells (PSCs) have been attracting exceptional interest among scientists since 2009, when they were first reported in the literature as light-energy converting devices with an efficiency of 3.8%.^1^ Currently, their record power conversion efficiency (PCE) has reached 25.8%,^2^ demonstrating their potential as the fastest-advancing solar cells and proving their competitiveness with the most established inorganic materials such as silicon, CIGS and CdTe.^3^ An astonishing performance of PSCs is a result of excellent optical and electrical properties of halide perovskites, such as broad absorption range and high absorption coefficient,^4^ high carrier mobility, long charge carrier diffusion length and lifetime.^5,6^ Moreover, PSCs demonstrate additional appealing features such as uncomplicated manufacturing, light weight and low cost.^7^

However, there are several issues in both types of PSCs, n-i-p and p-i-n structured, regarding the efficiency, long-term stability and manufacturing cost of devices. For conventional PSCs, where hole transporting material (HTM) is deposited on top of the perovskite layer, one of the main advantage is that organic semiconductors in general require milder processing conditions in comparison to inorganic counterparts.^8,9^ However, the oxidative doping process, which is used to match the indispensable electrical conductivity, and slow morphological degradation of HTMs have a substantial negative effect on the long-term stability of devices.^10,11^ As an alternative, p-i-n structured PSCs are not affected by aforementioned drawback since dopants are unnecessary for hole transporting layers (HTLs) in such device architecture. Furthermore, inverted PSCs allure researchers not only because of negligible *J*–*V* hysteresis effect,^12^ but also because of the simplicity of their manufacturing,^13^ which enables new possibilities for their application in tandem solar cells.^14^ On the other hand, since perovskite layer is solution-processable from a mixture of polar dimethylformamide:dimethyl sulfoxide (DMF:DMSO) solvents the selection of suitable HTMs is significantly reduced. Hence, up to now polymers such as poly(3,4-ethylenedioxythiophene)polystyrene sulfonate (PEDOT:PSS), poly(triarylamine) (PTAA) and poly[3-(4-carboxybutyl)thiophene-2,5-diyl] (P3CT) are often chosen as organic HTLs for p-i-n structured devices.^15^ However, polymers are not ideal HTMs in terms of difficult synthesis and tedious purification procedures. As an alternative, small organic molecules with a capability to be *in situ* polymerized into solvent-resistant 3D networks have been recently reported, demonstrating relatively high performances in inverted PSCs.^16^

Fluorene derivatives are important building blocks for HTMs in organic light-emitting diodes,^17^ organic thin-film transistors,^18^ dye-sensitized solar cells^19^ and, recently, PSCs with a reported PCE exceeding 17%.^20^ Wide applicability is reasoned by excellent hole drift mobility and simple functionalization of the molecule with a variety of introduced different groups, enabling fine-tuning of electrical and optical properties of HTMs. Additionally, fluorene is known for its relatively low cost, thus fluorene derivatives are excellent candidates for the synthesis of hole transporters, at the same time increasing the chances of commercial application of PSCs.^21^

In this study, thermally cross-linkable fluorene-based HTMs, functionalized with two vinylbenzyl groups, were synthesized and investigated. As a result of thermal polymerization these HTLs demonstrate good solvent resistance. Also, the cross-linking process has a positive effect on the hole drift mobility values of the compounds. Moreover, novel polymerized materials have proven to have more successful application in inverted architecture PSCs than compared to unpolymerized layers, which demonstrates the potential of dopant-free cross-linkable HTMs.

## 2. Results and discussion

**Scheme 1:**
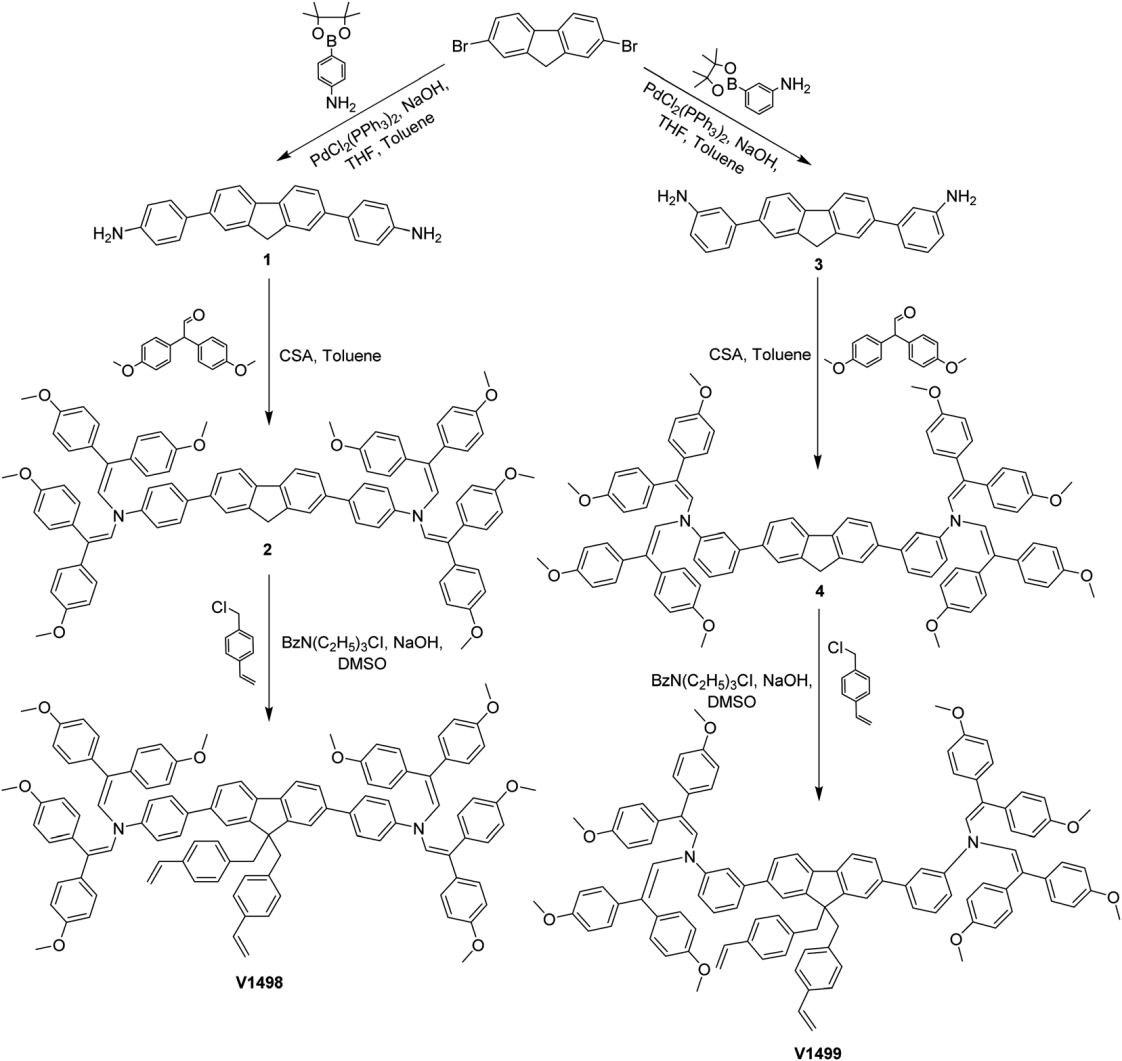
The synthetic route of fluorene-based HTMs.

The synthetic route to obtain cross-linkable compounds is shown in Scheme 1. 2,7-Dibromofluorene was chosen as a starting material for both final compounds V1498 and V1499 because of its commercial availability and relatively low price. During the first step, Suzuki reaction of the starting compound and either 4-aminophenylboronic acid pinacol ester or 3-aminophenylboronic acid pinacol ester were used to synthesize precursors 1 and 3, respectively. Afterwards, aminated-precursors were condensed with 2,2-bis(4-methoxyphenyl)acetaldehyde, following previously published procedure,^22^ to isolate intermediate compounds 2 and 4. During the third step, a simple alkylation reaction with 4-vinylbenzylchloride was used to generate 75% of the target product V1498 and 64% of V1499. Novel structures were fully characterized by nuclear magnetic resonance (NMR) spectroscopy, elemental analysis (EA), and mass spectrometry (MS) techniques. Detailed synthesis procedures are described in the ESI.†

The synthesis cost estimations of V1498 and V1499 have been performed following the procedure established by Osedach *et al.* (Table S1 and S2 in the ESI†).^23^ The material price of 1 g of either HTM (V1498 – 57€ per g, V1499 – 101€ per g) is significantly lower than the cost of 1 g of the most frequently used polymers (PTAA – 3040€ per g, P3CT – 500€ per g). Therefore, the production cost of perovskite solar cells should decrease accordingly.

**Fig. 1:**
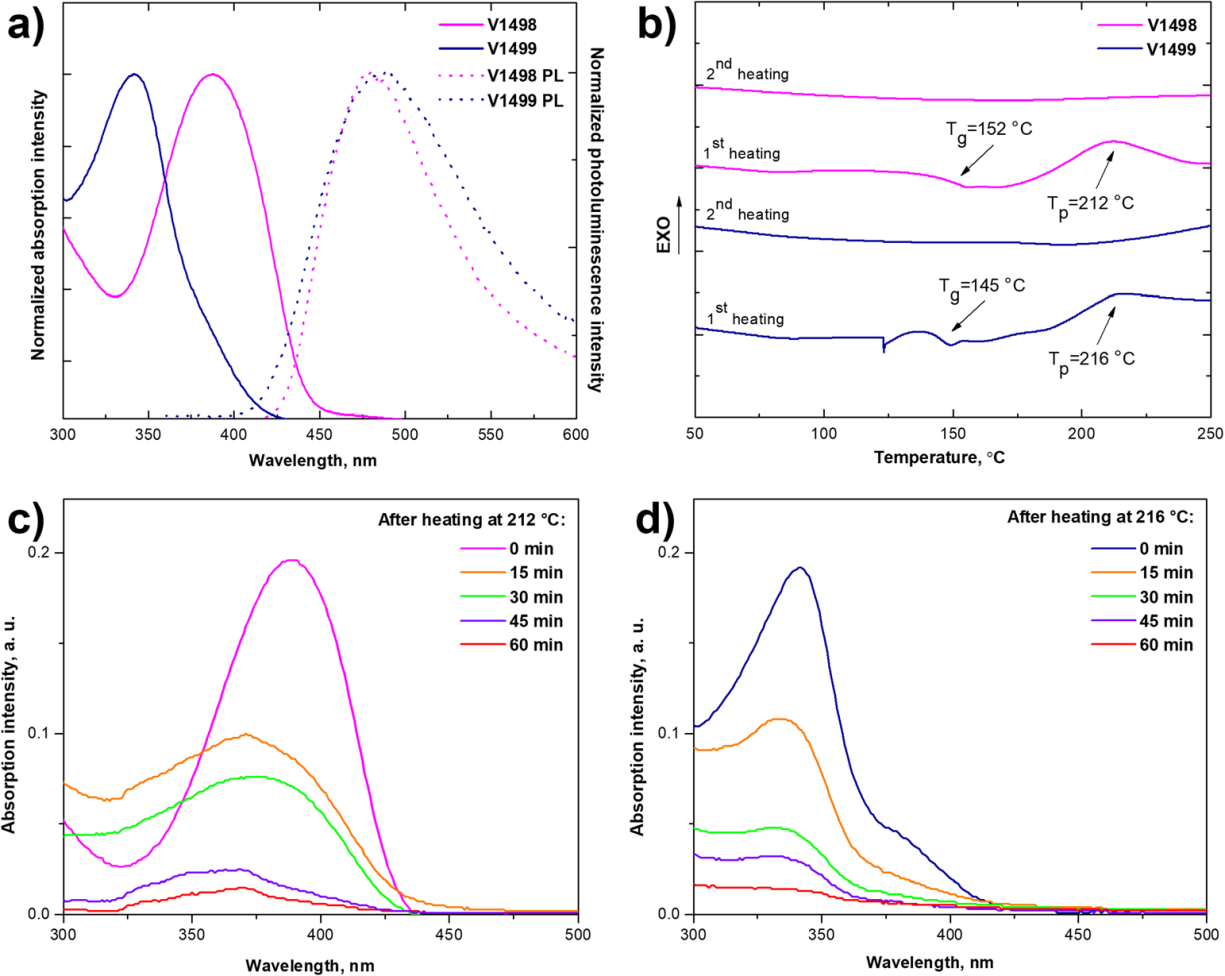
(a) UV-vis absorption (solid line) and photoluminescence (dashed line) spectra of thin films of V1498 and V1499; (b) DSC first and second heating curves of HTMs (scan rate 5 °C min^−1^, N_2_ atmosphere); cross-linking experiment of V1498 (c) and V1499 (d) films. The absorption of the solutions, prepared by dipping spin-coated and cured at adequate temperatures HTM films into THF for the respective duration.

To evaluate optical properties of newly synthesized fluorene-based compounds ultraviolet-visible (UV-vis) absorption and photoluminescence (PL) spectra were measured from the thin films. The spectra are shown in Fig. 1 and the pertinent data are summarized in Table 1. The absorption maximum (*λ*_max_) of V1498 was observed at 388 nm, while a hypsochromic shift of around 50 nm is observed for the compound V1499 (*λ*_max_ = 342 nm) due to the reduced π-conjugated system and differently substituted fluorene arms, which increase steric hindrance, therefore phenyl rings become more twisted out of the plane. After the cross-linking process the absorption maximum of both HTMs have shifted hypsochromically (*λ*_max_ = 343 nm for V1498, *λ*_max_ = 328 nm for V1499). PL spectra have shown that the emission maximum of V1498 and V1499 is at 478 and 488 nm, respectively, with large Stokes shift values of 90 nm (V1498) and 146 nm (V1499), which indicate that molecules can undergo large geometrical changes when excited, improving pore-filling of HTM.^24^ The intersection of absorption and emission spectra was used to calculate the optical gap (*E*_g_) of thin films and were estimated to be similar for both compounds, V1498 and V1499, respectively, at 2.84 and 3.00 eV (Fig. 1).

**Table 1:** Thermal, optical and photophysical properties of fluorene-based compounds

ID	*T*_g_ a (°C)	*T*_p_ a (°C)	*T*_dec_ a (°C)	*λ*_max_ b (nm)	*I*_P_ c (eV)	*E*_g_ d (eV)	*E*_ea_ e (eV)	*μ* f (1 × 10^6^ V cm^−1^) (cm^2^ V^−1^ s^−1^)	*μ*_0_ g (cm^2^ V^−1^ s^−1^)
V1498	152	212	395	261; 388	5.32	2.84	2.48	1.6 × 10^−3^	5 × 10^−5^
V1498 cross-linked					5.26		2.42	8 × 10^−3^	2 × 10^−4^
V1499	145	216	410	261; 342	5.49	3.00	2.49	2.3 × 10^−4^	1.5 × 10^−6^
V1499 cross-linked					5.43		2.43	6 × 10^−4^	3 × 10^−5^

a Polymerization (*T*_p_) and glass transition (*T*_g_) temperatures determined by DSC, scan rate 5 °C min^−1^; N_2_ atmosphere. Decomposition (*T*_dec_) temperatures observed from TGA, respectively (10 °C min^−1^, N_2_ atmosphere).

b Absorption spectra were measured of thin films.

c Ionization potentials of the films measured using PESA.

d *E*_g_ estimated from the intersection of absorption and emission spectra of solid films.

e *E*_ea_ = *I*_P_ − *E*_g_.

f Mobility value at strong electric fields.

g Mobility value at zero field strength.

To evaluate thermal stability of HTMs and to study the cross-linking process, thermogravimetric analysis (TGA) and differential scanning calorimetry (DSC) were used. Both fluorene-based compounds showed high thermal decomposition temperatures (*T*_dec_) corresponding to 5% weight loss, with a *T*_dec_ of 395 °C for V1498 and 410 °C for V1499, as shown in TGA analysis (Fig. S1, ESI†) and summarized in Table 1. DSC analysis demonstrates that both HTMs are fully amorphous since there were no endothermic peaks detected during both heating cycles (Fig. 1). During the first scan of V1498 the glass transition (*T*_g_) process was observed at 152 °C, followed by an exothermic peak at 212 °C corresponding to a thermal polymerization process. During the second scan there were no phase transitions detected, which indicates that the formation of cross-linked polymer was successful. During the first heating cycle of V1499 a slightly lower *T*_g_ of 145 °C was observed, followed by a polymerization peak at 216 °C, while no distinct transitions could be found on the second scan heating up to 260 °C.

Fluorene-based HTLs' ability to form cross-linked networks was evaluated by measuring the optical absorption before and after rinsing of the spin-coated film, by using UV-vis spectroscopy (detailed cross-linking procedure can be found in the ESI†). The results are demonstrated in Fig. 1. After annealing the films of V1498 and V 1499 at 212 °C and 216 °C, respectively, for 15 minutes the absorbance of the rinsed solutions diminishes, which indicates that the films are only partially cross-linked under these conditions. Therefore, a longer time frame was used to quantitatively cross-link the films. The cross-linking process for both monomers was completed after annealing for roughly 60 minutes. As an additional verification of a complete cross-linking process and the conversion of vinyl groups, Fourier-transform infrared spectra were recorded (Fig. S5, ESI†). After thermal polymerization the characteristic peaks of the vinyl groups at the 990–991 cm^−1^ and 904–906 cm^−1^ disappeared, which confirms a complete cross-linking reported previously.^25,26^

**Fig. 2:**
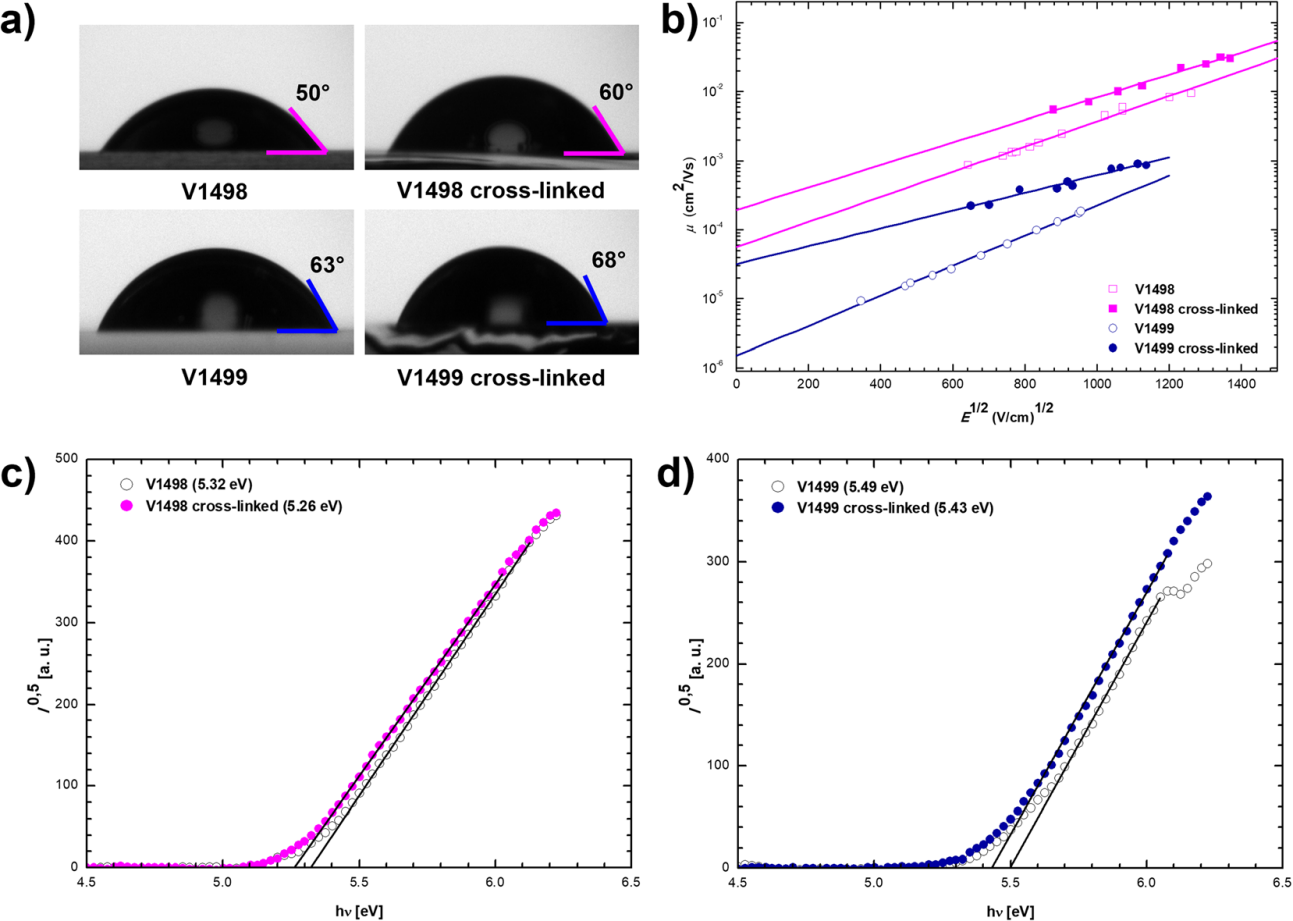
(a) Water contact angles of V1498 and V1499 before and after the cross-linking process; (b) electric field dependencies of hole drift mobilities in films of V1498 and V1499 before and after the cross-linking procedure; (c) and (d) photoemission in air spectra of charge transporting layers of V1498 and V1499 before and after the cross-linking procedure.

Contact angle measurements were carried out to evaluate surface properties of neat and cross-linked films. As demonstrated in Fig. 2, contact angles of water droplets on the annealed films increased around 10° when compared to contact angles on uncross-linked films. Moreover, a comparison between neat perovskite layer (Fig. S2†) and HTLs showed that HTMs have at least 15° larger contact angle than pristine perovskite. Therefore, it is expected that cross-linked HTLs with an improved hydrophobicity would result in an increased device stability.

To study charge transport properties of the novel HTMs xerographic time-of-flight (XTOF) technique was used (Fig. 2), the results are presented in Table 1. Compound V1498 demonstrates excellent hole drift mobility of 1.6 × 10^−3^ cm^2^ V^−1^ s^−1^ at strong electric fields. Sterically hindered compound V1499 displays one order of magnitude lower charge transporting properties, although still comparable to those of other prominent HTMs used in PSCs.^27,28^ Moreover, the hole mobilities of cross-linked films were also measured, the results indicate that charge transport properties of both compounds largely improve, which might be a result of the enhanced intermolecular packing caused by the cross-linking process.

The solid-state ionization potentials (*I*_P_) of fluorene-based HTMs were evaluated by using the photoelectron spectroscopy in air (PESA). As shown in Fig. 2, the highest occupied molecular orbital (HOMO) energy level of V1498 and V1499 was demonstrated to be −5.32 and −5.49 eV, respectively. As expected, the cross-linking process only had a negligible effect on these values by reducing the *I*_P_ of both compounds by 0.06 eV. The lowest unoccupied molecular orbital (LUMO), calculated from the *I*_P_ and *E*_g_, is 2.48 and 2.49 eV for not cross-linked V1498 and V1499, respectively (Table 1). HOMO and LUMO energy levels of these HTMs are suitable for the application in PSCs.^29,30^

**Fig. 3:**
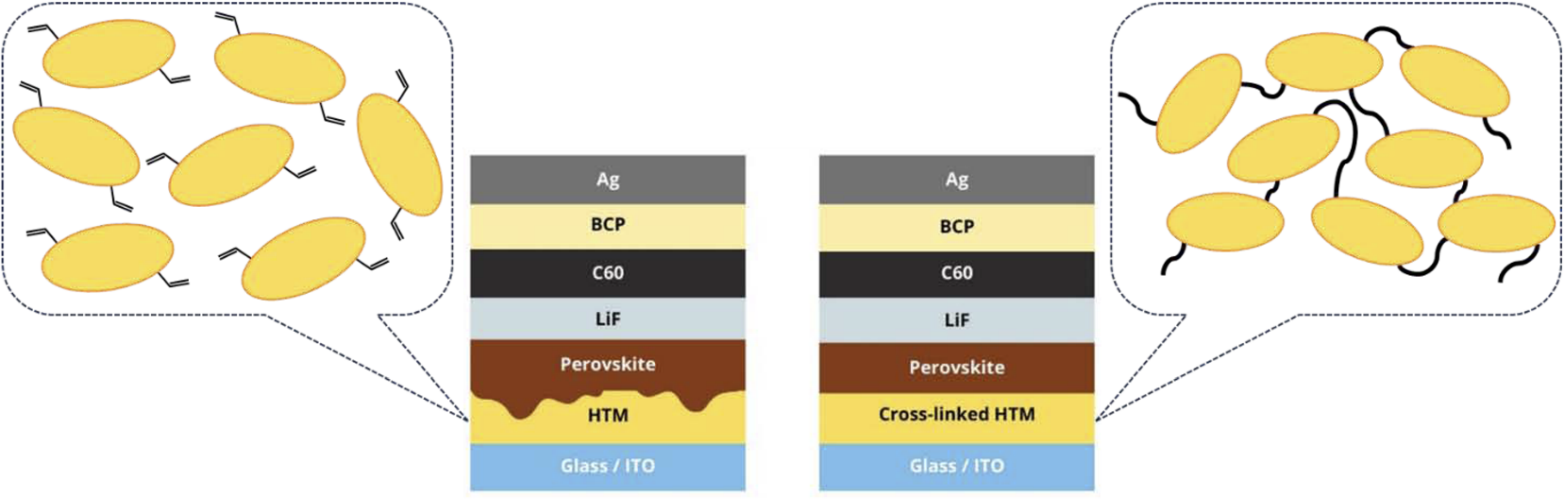
Two solar cell structures used in this study.

To evaluate the efficacy of synthesized compounds acting as HTLs in PSCs, inverted p-i-n structured devices were fabricated and characterized having the structure of: an indium tin oxide (ITO)-coated glass substrate/HTM/perovskite/LiF/C_60_ as electron transport layer (ETL)/bathocuproine (BCP)/a silver electrode (Fig. 3). Triple-cation perovskite^31^ was used as an absorbing layer with a nominal precursor solution composition of Cs_0.05_(FA_0.83_MA_0.17_)_0.95_Pb(I_0.83_Br_0.17_)_3_. The organic HTLs were deposited on ITO by spin-coating their toluene solution. For the cross-linking, substrates were annealed in a nitrogen atmosphere at 212 °C for 1 h for V1498 and at 216 °C for 1 h for V1499. The detailed information for PSC fabrication is provided in the ESI.†

**Fig. 4:**
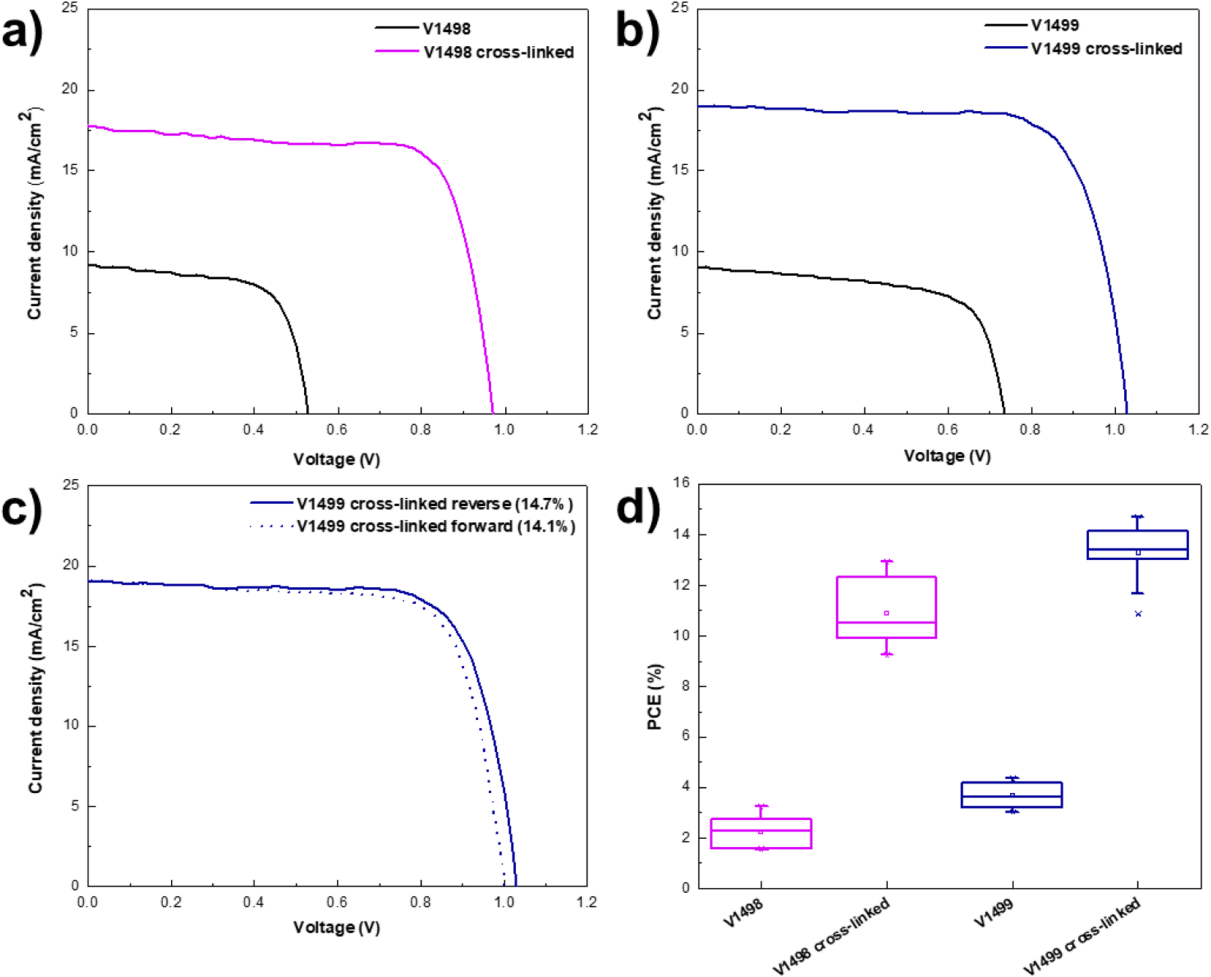
*J*/*V* measurements of the champion PSCs prepared with V1498 (a) and V1499 (b) before and after cross-linking, reverse scan; (c) comparison of *J*/*V* curves of cross-linked V1499, demonstrating reverse and forward scans; (d) statistical distribution of PCE values of PSCs with new HTMs, before and after cross-linking.

The photocurrent density–voltage (*J*/*V*) characteristics were used to evaluate the photovoltaic performance of the fabricated devices. Both devices containing monomer films demonstrated low open-circuit voltages (*V*_OC_) of 688 mV for V1498 and 735 mV for V1499 of best performing devices, as shown in Fig. 4 and Table 2. Lower *V*_OC_ values can be attributed to an increased interfacial recombination.^32^ As expected, deeper HOMO level of V1499 results in a higher *V*_OC_ value^33^ when compared to V1498. Additionally, the PSCs with the neat films were compared with the devices containing thermally cross-linked HTLs to assess the impact of the cross-linking process on the device performance. *V*_OC_ values of devices containing V1498 and V1499 were significantly enhanced after annealing up to 1029 mV. The same trend can be observed for the fill factor (FF), where values after the cross-linking process increased from approximately 67 to 75% for champion devices. As demonstrated in Fig. 4, PCE of PSCs containing neat films was by 80% (for V1498) on average lower compared to the devices with cross-linked HTLs. Only a small hysteresis was recorded (Fig. 4). Significantly improved device performance (from 4.4 to 14.7% for V1499) while using the annealed HTMs could be attributed to the higher FF and hole drift mobility's values,^34^ therefore, a better charge transport through the film. The results are also in agreement with the UV-vis absorption spectra. The reference device with Me-4PACz HTM showed a maximum efficiency of 20%.

**Table 2:** Photovoltaic parameters of the studied PSCs using different HTMs. The data derived from *J*/*V* scans (reverse and forward), and includes average standard deviation, as well as the best performance parameters (in brackets)

HTM^a^	*J*_sc_ b (mA cm^−2^)	*V*_oc_ b (mV)	FF^b^ (%)	PCE^b^ (%)
V1498	8.2 ± 0.5 (9.2)	585 ± 57 (688)	46.6 ± 9.7 (66.7)	2.2 ± 0.6 (3.2)
V1498 cross-linked	16.0 ± 1.2 (17.7)	946 ± 13 (972)	71.9 ± 2.6 (74.9)	10.9 ± 1.2 (12.9)
V1499	9 ± 0.2 (9.3)	686 ± 40 (735)	59.5 ± 5.8 (66.8)	3.7 ± 0.5 (4.4)
V1499 cross-linked	18.5 ± 0.5 (19.1)	1001 ± 16 (1029)	71.9 ± 5.2 (75.4)	13.3 ± 1.1 (14.7)
Me-4PACz	21.5 ± 0.3 (21.9)	1126 ± 3 (1129)	80.6 ± 0.6 (81.6)	19.5 ± 0.3 (20.0)

a Concentration of 1.5 mg ml^−1^ was used for fluorene-based HTMs.

b The average and standard deviation values were calculated from 6 devices.

## 3. Conclusions

To summarize, two new fluorene-based HTMs were designed and synthesized. Both compounds contain two vinyl groups, therefore they can undergo thermal cross-linking process, which proceeds at ∼214 °C for 1 h. The cross-linked HTLs demonstrate higher hole drift mobilities, increased FF and open-circuit voltage values when compared to the devices containing neat films. As a result, p-i-n structured PSCs with thermally polymerized layers improve PCE by 80% on average, demonstrating a great promise of the proposed strategy.

## Conflicts of interest

There are no conflicts to declare.

## Supplementary Material

RA-013-D3RA03492E-s001
