# Supplementary material for: Thermally cross-linkable fluorene-based hole transporting materials: synthesis, characterization, and application in perovskite solar cells
Source: RSC Adv. 2023 Sep 8;13(38):26933–9. doi: 10.1039/d3ra03492e (PMC10485655; doi:10.1039/d3ra03492e)
Supplement: RA-013-D3RA03492E-s001 [file RA-013-D3RA03492E-s001.pdf]

Electronic Supplementary Material (ESI) for RSC Advances.
This journal is © The Royal Society of Chemistry 2023

Supporting Information

Thermally Cross-linkable Fluorene-Based Hole-Transporting Materials: Synthesis, Characterization, and Application in Perovskite Solar Cells

Deimante Vaitukaityte,[1] Artiom Magomedov,[1,2] Kasparas Rakstys,[1] Simon Kwiatkowski,[3] Egidijus Kamarauskas,[4] Vygintas Jankauskas,[4] Jolanta Rousseau,[3] Vytautas Getautis,[1]*

[1]Department of Organic Chemistry, Kaunas University of Technology, Radvilenu pl. 19, Kaunas 50254, Lithuania

[2]Department of Chemical and Biological Engineering, University of Colorado, Boulder, CO, 80309, USA

[3]Univ. Artois, CNRS, Centrale Lille, Univ. Lille, UMR 8181–UCCS–Unité de Catalyse et Chimie du Solide, Faculty of Science Jean Perrin, Rue Jean Souvraz SP 18, F-62300 Lens, France

[4]Institute of Chemical Physics, Vilnius University, Sauletekio al. 3, Vilnius 10257, Lithuania

*Corresponding author:

vytautas.getautis@ktu.lt

TABLE OF CONTENTS

General Methods and Materials 3

Ionization Potential Measurements 3

Hole Drift Mobility Measurements 4

Cross-linking Experiment 4

Fabrication of Perovskite Solar Cells 5

Detailed Synthetic Procedures 6

Thermal Properties 12

Water contact angle 12

Illustration of 3D network polymer 12

UV−Vis spectra 13

FT-IR spectroscopy 13

NMR spectra 14

Synthesis cost 16

References 18

General Methods and Materials

Chemicals required for the synthesis were purchased from Sigma-Aldrich and TCI Europe, and used as received without additional purification. 2,2-bis(4-methoxyphenyl)acetaldehyde was synthesized following the literature procedure.[1] ^{1}H NMR spectra were recorded at 400 MHz on a Bruker Avance III spectrometer with a 5 mm double resonance broad band BBO z-gradient room temperature probe, ^{13}C NMR spectra were collected using the same instrument at 101 MHz. The chemical shifts, expressed in ppm, were relative to tetramethylsilane (TMS). All the NMR experiments were performed at 25 °C. Reactions were monitored by thin-layer chromatography on ALUGRAM SIL G/UV254 plates and developed with UV light. Silica gel (grade 9385, 230–400 mesh, 60 Å, Aldrich) was used for column chromatography. Elemental analysis was performed with an Exeter Analytical CE-440 elemental analyzer, Model 440 C/H/N/. MS were recorded on Waters SQ Detector 2 Spectrometer using electrospray ionization (ESI) technique.

Thermogravimetric analysis (TGA) was performed on a Q50 thermogravimetric analyzer (TA Instruments) at a scan rate of 10 °C min^{-1} under nitrogen atmosphere. The values are given for a weight-loss of 5% (T_{dec}). Differential scanning calorimetry (DSC) was performed on a TA Instruments Q2000 differential scanning calorimeter under nitrogen atmosphere. Heating and cooling rate is 5 °C min^{-1}.

UV–vis spectral analysis of thin films was performed on a Perkin Elmer Lambda 35 UV–VIS spectrophotometer. Diffraction grating crack width is 2 nm. Spectral recording speed is 2 nm s^{-1}. The wavelength λ is given in nm. The time-resolved fluorescence spectra of thin films were recorded on an Edinburgh Instruments FLS920 light emission intensity spectrophotometer.

Ionization Potential Measurements

The solid state ionization potential (I_p) of the layers of the synthesized compounds was measured by the electron photoemission in air method;[2,3] the measurement error is evaluated as 0.03 eV. The samples for the ionization potential measurement were prepared by dissolving materials in THF and were coated on Al plates pre-coated with ~0.5 μm thick methylmethacrylate and methacrylic acid copolymer adhesive layer. The thickness of the transporting material layer was 0.5-1 μm. Usually photoemission experiments are carried out in vacuum and high vacuum is one of the main requirements for these measurements. If vacuum is not high enough the sample surface oxidation and gas adsorption are influencing the measurement results. In our case, however, the organic

materials investigated are stable enough to oxygen and the measurements may be carried out in the air. The samples were illuminated with monochromatic light from the quartz monochromator with deuterium lamp. The power of the incident light beam was (2-5)·10^{-8} W. The negative voltage of –300 V was supplied to the sample substrate. The counter-electrode with the 4.5×15 mm^2 slit for illumination was placed at 8 mm distance from the sample surface. The counter-electrode was connected to the input of the BK2-16 type electrometer, working in the open input regime, for the photocurrent measurement. The 10^{-15} – 10^{-12} A strong photocurrent was flowing in the circuit under illumination. The photocurrent I is strongly dependent on the incident light photon energy hν. The $I^{0.5} = f(h\nu)$ dependence was plotted. Usually, the dependence of the photocurrent on incident light quanta energy is well described by linear relationship between $I^{0.5}$ and $h\nu$ near the threshold. The linear part of this dependence was extrapolated to the $h\nu$ axis and I_p value was determined as the photon energy at the interception point.

Hole Drift Mobility Measurements

The samples for the hole mobility measurements were prepared by spin-coating the solutions of the compositions of investigated compounds on glass plates with conductive Al layer. The layer thickness was in the range of 1.8-2.8 µm. The hole drift mobility was measured by xerographic time of flight technique (XTOF).[4–6] Electric field was created by positive corona charging. The charge carriers were generated at the layer surface by illumination with pulses of nitrogen laser (pulse duration was 1 ns, wavelength 337 nm). The layer surface potential decrease as a result of pulse illumination was up to 1-5 % of initial potential before illumination. The capacitance probe that was connected to the wide frequency band electrometer measured the speed of the surface potential decrease dU/dt. The transit time t_t was determined by the kink on the curve of the dU/dt transient in double logarithmic scale. The drift mobility was calculated by the formula $\mu = d^2/U_0 t_t$, where d is the layer thickness, U_0 – the surface potential at the moment of illumination.

Cross-linking Experiment

The glass substrate was cleaned by sonication in TICKOPUR R 30 neutral cleaner (3 %) and subsequently rinsed in distilled water and acetone. For the spin-coating solutions of HTMs in chlorobenzene (80 mg ml^{-1}) were prepared. They were further spin-coated on pre-cleaned glass substrates at 4000 rpm for 30 s. The resulting films were heated on a hot plate in the air atmosphere

at a temperature of at 212 °C ± 5 °C for **V1498** and 216 °C ± 5 °C for **V1499** (as determined from DSC measurements), for different periods of time (0, 15, 30, 45, 60 minutes). After cooling, films were separately dipped into tetrahydrofuran (5 ml) for 15 minutes. Resulting solutions were further used to record UV/vis spectra to evaluate the amount of the washed HTM and consequently polymerization conversion efficiency.

Fabrication of Perovskite Solar Cells

ITO substrates were pre-cleaned by consequentially sonicating for 15 min in Extran detergent, deionized water, acetone, and 2-propanol. After that, substrates were cleaned by UV-ozone for 15min. All the next steps were performed in the N_2-filled glovebox. HTMs were deposited from the 1.5 mg/ml solutions in Toluene by dynamic spin-coating at 6000 rpm (3 s ramp) for 30 s. For the cross-linking, the substrates were annealed at 212 °C (60 min) for **V1498** and at 216 °C (60 min) for **V1499**. The SAM solution (4:1 v:v of 1 mM/l Me-4PACz+6dPA in ethanol) was spin coated at 3000 rpm for 10s, after which the substrate was anealed at 100°C for 10 min.[7] After that, perovskite solution (507 mg PbI_2, 73.4 mg $PbBr_2$, 172 mg FAI, 22.4 mg MABr dissolved in 0.8 ml DMF and 0.2 ml DMSO, followed by the addition of 40 µl of stock CsI solution (390 mg CsI in 1 ml DMSO)) was statically spin-coated at 5000 rpm (1.7 s ramp) for 35 s, followed by dispensing 300 µl of Anisole after 10 s of spinning program. After spin-coating the substrates were annealed at 100°C for 1h. The device was finalized by evaporation of the LiF (1 nm), C60 (23 nm), BCP (8 nm) and Ag (100 nm).

Detailed Synthetic Procedures

4,4'-(9*H*-fluorene-2,7-diyl)dianiline (1)

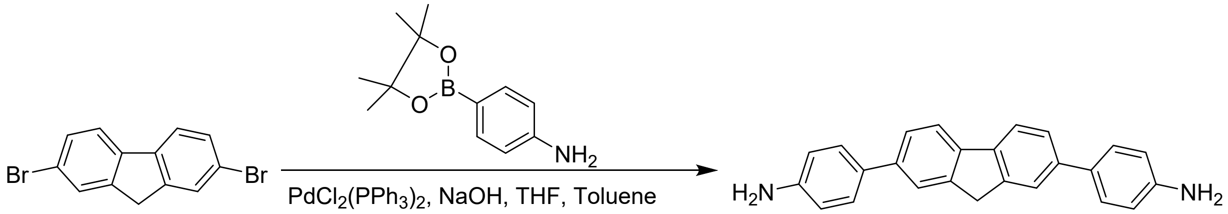

To a suspension of 2,7-dibromofluorene (1.00 g, 3.09 mmol) and 4-aminophenylboronic acid pinacol ester (1.35 g, 6.17 mmol) in 80 mL of mixed solvent of anhydrous tetrahydrofuran and anhydrous toluene (1:1), sodium hydroxide (1.85 g, 46.29 mmol) was added, followed by the addition of $PdCl_2(PPh_3)_2$ (0.54 g, 0.77 mmol). The mixture was degassed with argon for three times and was refluxed at 95 °C for 23 h under argon. After termination of the reaction (TLC, THF:*n*-hexane, 12:13) the reaction mixture was filtered through celite and celite was washed with THF. After filtration solvents were evaporated. The crude product was crystallized from THF/ethanol 1:1 (80 ml); filtered off and washed with ethanol for two times (0.60 g; 56%). ^{1}H NMR (400 MHz, DMSO-d_6) δ: 7.83 (d, J = 8.0 Hz, 2H); 7.74 (s, 2H); 7.55 (d, J = 8.0 Hz, 2H); 7.43 (d, J = 8.1 Hz, 4H); 6.66 (d, J = 8.1 Hz, 4H); 5.22 (s, 4H); 3.96 (s, 2H) ppm. ^{13}C NMR (101 MHz; DMSO-d_6) δ: 148.24; 143.79; 139.11; 138.77; 127.74; 127.17; 124.18; 122.00; 119.99; 114.28; 36.58 ppm. Anal. calcd for $C_{25}H_{20}N_2$: C 86.17; H 5.79; N 8.04; found: C 85.92; H 5.83; N 7.82. $C_{25}H_{20}N_2$ [M^+] exact mass = 348.16; MS (ESI) = 349.11.

4,4'-(9*H*-fluorene-2,7-diyl)bis(*N*,*N*-bis(2,2-bis(4-methoxyphenyl)vinyl)aniline) (2)

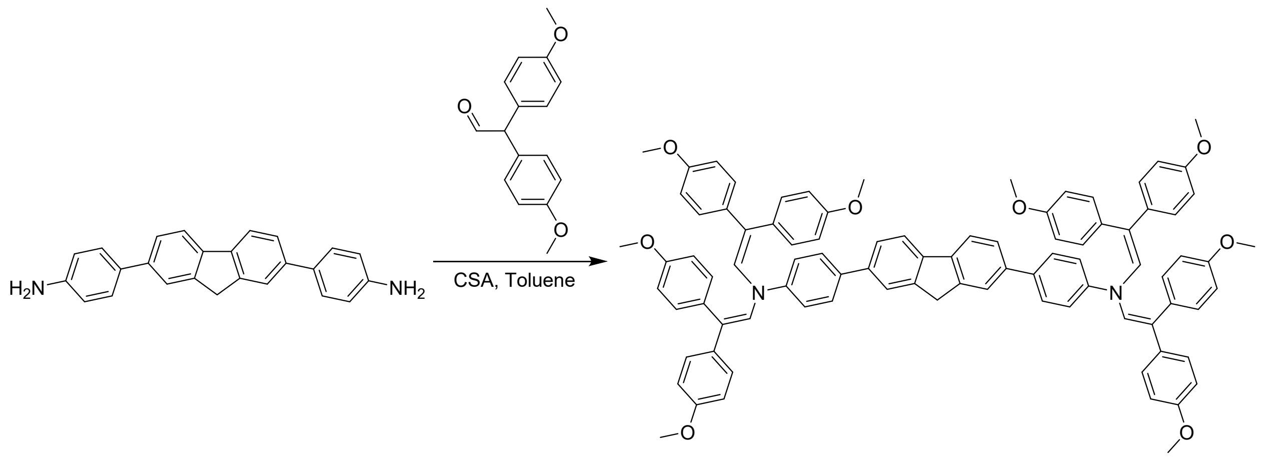

Compound **1** (0.20 g; 0.57 mmol) was dissolved in toluene (8 mL + volume of the Dean-Stark trap); (+/-)camphor-10-sulphonic acid (0.20 g; 0.86 mmol) was added and the mixture was heated at reflux for 20 minutes. Afterwards; 2;2-bis(4-methoxyphenyl)acetaldehyde (0.77 g; 2.99 mmol) was added; and reflux continued using a Dean-Stark trap. After termination of the reaction (5 h; TLC; THF:*n*-hexane; 2:3) the reaction mixture was poured into 15-fold excess of ethanol. The product was filtered off and washed with ethanol. The crude product was purified by column chromatography using THF:*n*-hexane (v:v; 6:19) eluent. The obtained product was precipitated from THF into 15-fold excess of ethanol. The precipitate was filtered off and washed with ethanol to collect compound **2** (0.44 g; 59% yield). ^{1}H NMR (400 MHz, THF-d_8) δ: 7.84 (d, J = 11.0 Hz, 4H); 7.65 (dd, J = 13.3, 8.1 Hz, 6H); 7.16 (d, J = 8.2 Hz, 4H); 7.01 (d, J = 8.3 Hz, 8H); 6.87 (d, J = 8.2 Hz, 8H); 6.62 (d, J = 8.3 Hz, 8H); 6.46 (d, J = 8.3 Hz, 8H); 5.82 (s, 4H); 4.01 (s, 2H); 3.84 (s, 12H); 3.71 (s, 12H) ppm. ^{13}C NMR (101 MHz; THF-d_8) δ: 160.58; 160.25; 146.55; 145.34; 141.25; 140.45; 135.51; 135.29; 133.77; 132.58; 131.61; 129.87; 128.63; 127.64; 126.28; 123.87; 120.94; 117.63; 114.88; 113.88; 55.75; 55.50; 37.86 ppm. Anal. calcd for $C_{89}H_{76}N_2$: C, 82.13; H 5.89; N 2.15; found: C 81.88; H 5.96; N 2.21. $C_{89}H_{76}N_2O_8$ [M^+] exact mass = 1300.56; MS (ESI) = 1301.17.

4,4'-(9,9-bis(4-vinylbenzyl)-9*H*-fluorene-2,7-diyl)bis(*N*,*N*-bis(2,2-bis(4-methoxyphenyl)vinyl)aniline) (V1498)

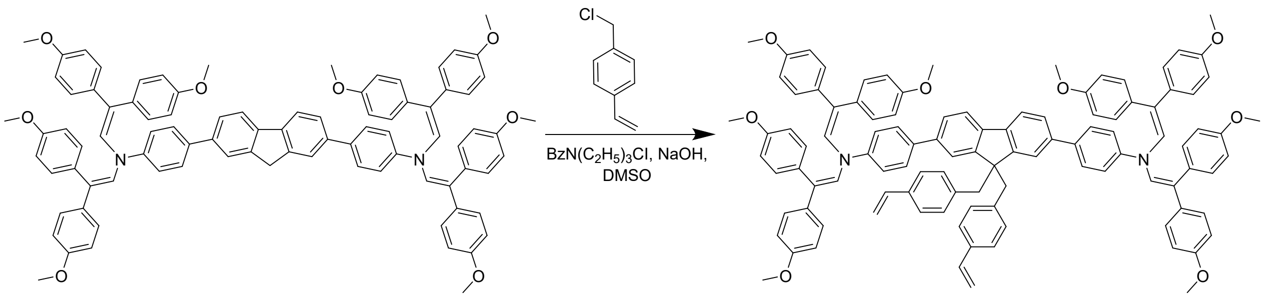

The solution of compound **2** (0.25 g, 0.19 mmol) was degassed in dimethyl sulfoxide (10 mL) with argon for 30 minutes. Afterwards, benzyltriethylammonium chloride (0.004 g, 0.02 mmol) and aqueous NaOH (50%, 0.08 mL) were added. When the reaction mixture turned red 4-vinylbenzylchloride (0.06 mL, 0.42 mmol) was added and the mixture was stirred at room temperature. After termination of the reaction (4 h, TLC, acetone:*n*-hexane, 7:18) the reaction mixture was extracted with ethyl acetate. The organic layer was dried over anhydrous Na_2SO_4, filtered and solvent evaporated. The crude product was purified by column chromatography using THF:*n*-hexane (v:v; 7:18) eluent. The obtained product was precipitated from THF into 15-fold excess of ethanol. The precipitate was filtered off and washed with ethanol to collect **V1498** as a yellow solid (0.22 g; 75% yield). ^{1}H NMR (400 MHz; THF-d_8) δ: 7.81 (s, 2H); 7.71 (d, J = 8.3 Hz, 4H); 7.47 (q, J = 8.0 Hz, 4H); 7.19 (d, J = 8.3 Hz, 4H); 7.03 (d, J = 8.4 Hz, 8H); 6.98 (d, J = 7.9 Hz, 4H); 6.89 (d, J = 8.2 Hz, 8H); 6.74 (d, J = 7.8 Hz, 4H); 6.64 (d, J = 8.3 Hz, 8H); 6.49 (d, J = 8.5 Hz, 10H); 5.84 (s, 4H); 5.53 (d, J = 17.6 Hz, 2H); 4.97 (d, J = 10.8 Hz, 2H); 3.85 (s, 12H); 3.72 (s, 12H); 3.52 (s, 4H) ppm. ^{13}C NMR (101 MHz; THF-d_8) δ: 160.49; 160.14; 150.27; 146.42; 140.53; 139.68; 138.34; 137.83; 136.15; 135.53; 135.12; 133.69; 132.57; 131.49; 131.36; 129.76; 128.56; 127.57; 126.38; 125.87; 123.39; 120.76; 117.49; 114.79; 113.78; 112.68; 58.03; 55.65; 55.38; 46.04 ppm. Anal. calcd for $C_{107}H_{92}N_2$: C 83.78; H 6.05; N 1.83; found: C 82.99; H 5.99; N 1.97. $C_{107}H_{92}N_2O_8$ [M$^+$] exact mass = 1532.69; MS (ESI) = 1533.46.

3,3'-(9*H*-fluorene-2,7-diyl)dianiline (3)

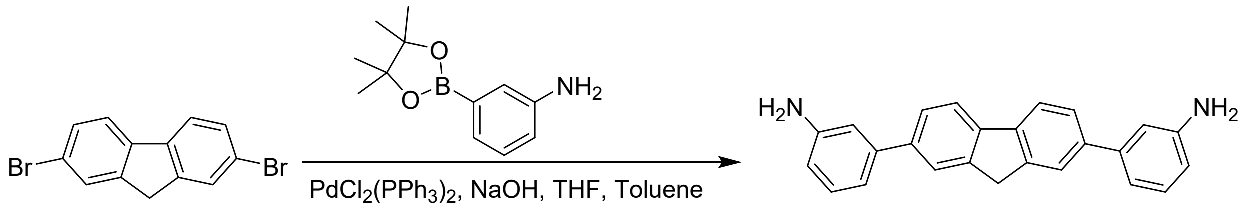

To a suspension of 2,7-dibromofluorene (1.00 g, 3.09 mmol) and 3-aminophenylboronic acid pinacol ester (1.35 g, 6.17 mmol) in 80 mL of mixed solvent of anhydrous tetrahydrofuran and anhydrous toluene (1:1), sodium hydroxide (1.85 g, 46.29 mmol) was added, followed by the addition of $PdCl_2(PPh_3)_2$ (0.54 g, 0.77 mmol). The mixture was degassed with argon for three times and was refluxed at 95 °C for 24 h under argon. After termination of the reaction (TLC, THF:*n*-hexane, 12:13) the reaction mixture was filtered through celite and celite was washed with THF. After filtration solvents were evaporated. The crude product was crystallized from THF/ethanol 1:1 (40 ml); filtered off and washed with ethanol for two times (0.70 g; 65%). ^{1}H NMR (400 MHz, DMSO-d_6) δ: 7.93 (d, J = 8.0 Hz, 2H); 7.78 (s, 2H); 7.59 (d, J = 8.0 Hz, 2H); 7.12 (t, J = 7.7 Hz, 2H); 6.93 (s, 2H); 6.86 (d, J = 7.6 Hz, 2H); 6.58 (d, J = 8.9 Hz, 2H); 5.16 (s, 4H); 4.02 (s, 2H) ppm. ^{13}C NMR (101 MHz, DMSO-d_6) δ: 149.13; 143.94; 141.14; 139.90; 139.70; 129.43; 125.36; 123.19; 120.27; 114.42; 113.07; 112.18; 36.59 ppm. Anal. calcd for $C_{25}H_{20}N_2$: C 86.17; H 5.79; N 8.04; found: C 86.07; H 5.70; N 7.74. $C_{25}H_{20}N_2$ [M^+] exact mass = 348.16; MS (ESI) = 349.05.

3,3'-(9*H*-fluorene-2,7-diyl)bis(*N*,*N*-bis(2,2-bis(4-methoxyphenyl)vinyl)aniline) (4)

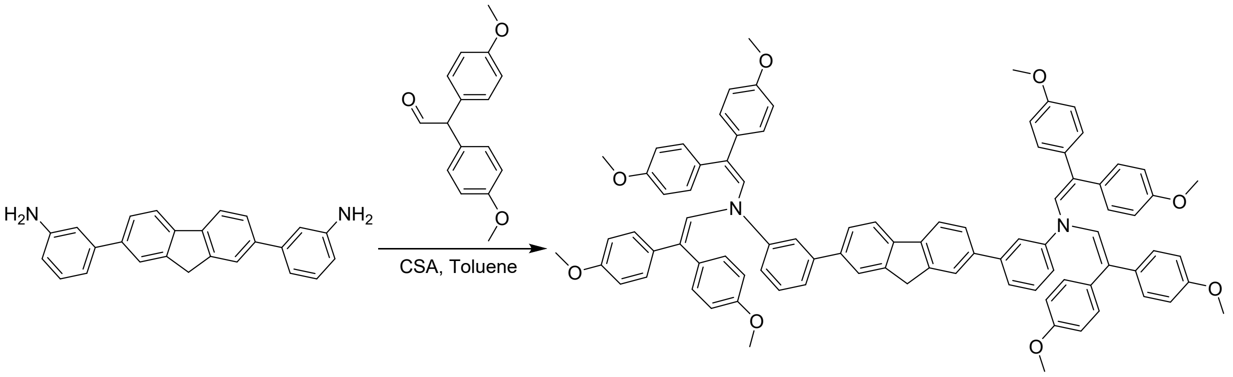

Compound **3** (0.25 g; 0.72 mmol) was dissolved in toluene (10 mL + volume of the Dean-Stark trap); (+/-)camphor-10-sulphonic acid (0.33 g; 1.42 mmol) was added and the mixture was heated at reflux for 20 minutes. Afterwards; 2;2-bis(4-methoxyphenyl)acetaldehyde (0.77 g; 2.99 mmol) was added; and reflux continued using a Dean-Stark trap. After termination of the reaction (5 h; TLC; THF:*n*-hexane; 2:3) the reaction mixture was poured into 15-fold excess of ethanol. The product was filtered off and washed with ethanol. The crude product was purified by column chromatography using THF:*n*-hexane (v:v; 7:18) eluent. The obtained product was precipitated from THF into 15-fold excess of ethanol. The precipitate was filtered off and washed with ethanol to collect compound **4** (0.33 g; 35% yield). ^{1}H NMR (400 MHz; THF-d_8) δ: 7.81 (d, J = 9.8 Hz, 4H); 7.60 (d, J = 7.9 Hz, 2H); 7.42 – 7.25 (m, 6H); 7.07 (d, J = 10.0 Hz, 2H); 7.01 (d, J = 8.3 Hz, 8H); 6.86 (d, J = 8.4 Hz, 8H); 6.62 (d, J = 8.3 Hz, 8H); 6.46 (d, J = 8.4 Hz, 8H); 5.86 (s, 4H); 3.94 (s, 2H); 3.83 (s, 12H); 3.70 (s, 12H) ppm. ^{13}C NMR (101 MHz; THF-d_8) δ: 160.44; 160.09; 147.72; 145.24; 143.86; 141.65; 141.03; 135.18; 133.70; 132.35; 131.51; 130.34; 129.78; 127.64; 127.01; 124.64; 121.20; 120.86; 116.17; 115.73; 114.73; 113.74; 55.62; 55.36; 37.64 ppm. Anal. calcd for $C_{89}H_{76}N_2$: C, 82.13; H 5.89; N 2.15; found: C 81.33; H 6.40; N 2.15. $C_{89}H_{76}N_2O_8$ [M^+] exact mass = 1300.56; MS (ESI) = 1301.22.

3,3'-(9,9-bis(4-vinylbenzyl)-9*H*-fluorene-2,7-diyl)bis(*N*,*N*-bis(2,2-bis(4-methoxyphenyl)vinyl)aniline) (V1499)

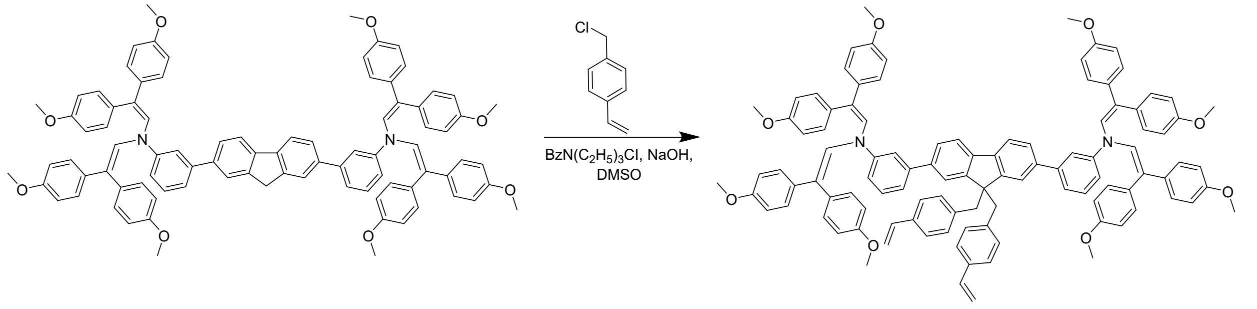

The solution of compound **4** (0.20 g, 0.15 mmol) was degassed in dimethyl sulfoxide (8.5 mL) with argon for 30 minutes. Afterwards, benzyltriethylammonium chloride (0.004 g, 0.02 mmol) and aqueous NaOH (50%, 0.06 mL) were added. When the reaction mixture turned red 4-vinylbenzylchloride (0.05 mL, 0.34 mmol) was added and the mixture was stirred at room temperature. After termination of the reaction (24 h, TLC, acetone:*n*-hexane, 6:19) the reaction mixture was extracted with ethyl acetate. The organic layer was dried over anhydrous Na_2SO_4, filtered and solvent evaporated. The crude product was purified by column chromatography using THF:*n*-hexane (v:v; 7:18) eluent. The obtained product was precipitated from THF into 15-fold excess of ethanol. The precipitate was filtered off and washed with ethanol to collect **V1499** as a yellow solid (0.15 g; 64% yield). ^{1}H NMR (400 MHz; THF-d_8) δ: 7.80 (s, 2H); 7.50 – 7.30 (m, 10H); 7.07 (dd, J = 18.2, 8.1 Hz, 10H); 6.86 (dd, J = 17.2, 8.1 Hz, 12H); 6.62 (d, J = 8.0 Hz, 12H); 6.49 (d, J = 8.4 Hz, 8H); 6.39 (dd, J = 17.7, 10.9 Hz, 2H); 5.90 (s, 4H); 5.46 (d, J = 17.6 Hz, 2H); 4.96 (d, J = 10.9 Hz, 2H); 3.84 (s, 12H); 3.70 (s, 12H); 3.47 (s, 4H) ppm. ^{13}C NMR (101 MHz; THF-d_8) δ: 160.45; 160.10; 150.27; 147.71; 143.98; 141.19; 140.48; 138.21; 137.78; 136.08; 135.21; 133.71; 132.37; 131.53; 131.05; 130.34; 129.81; 127.72; 127.29; 125.88; 124.21; 121.23; 120.75; 116.18; 115.86; 114.75; 113.77; 112.63; 58.10; 55.63; 55.35; 46.12 ppm. Anal. calcd for $C_{107}H_{92}N_2$: C 83.78; H 6.05; N 1.83; found: C 82.83; H 6.13; N 1.82. $C_{107}H_{92}N_2O_8$ [M$^+$] exact mass = 1532.69; MS (ESI) = 1533.16.

Thermal Properties

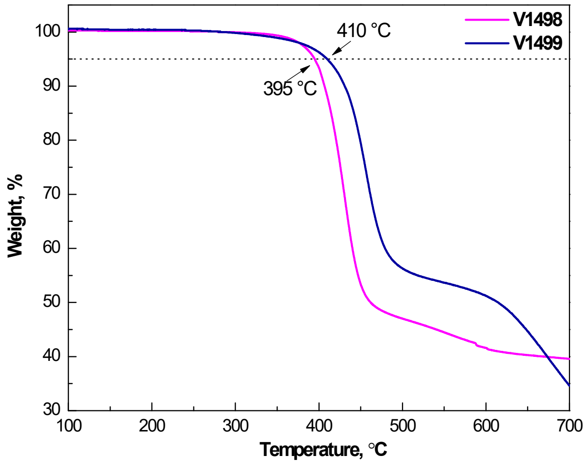

Figure S1. Thermogravimetric analysis (TGA) data (heating rate of 10 °C/min, N_2 atmosphere).

Water contact angle

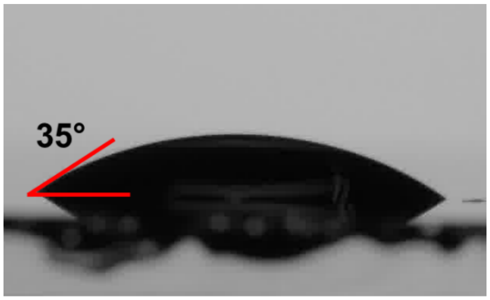

Figure S2. Water contact angle of perovskite.

Illustration of 3D network polymer

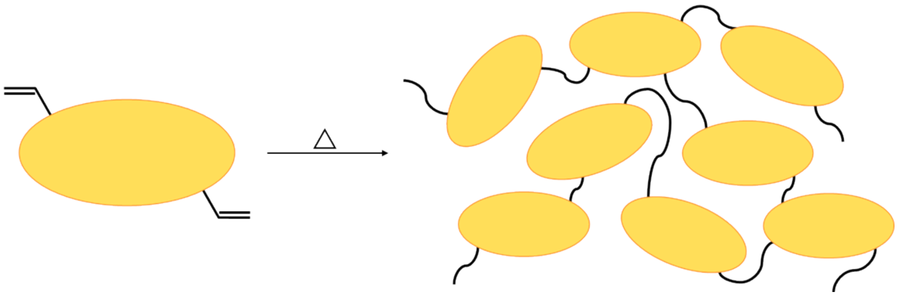

Figure S3. A schematic illustration of a 3D network polymer formation during thermal polymerization.

UV−Vis spectra

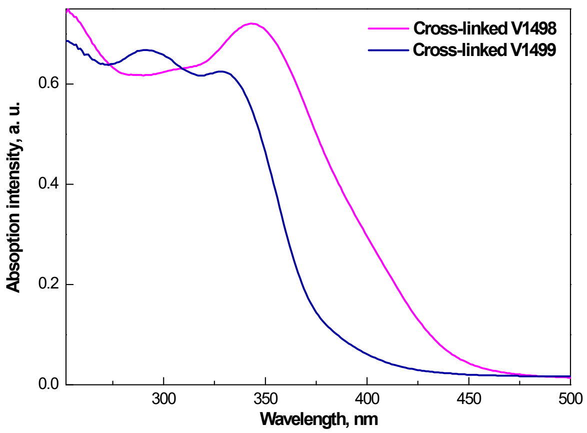

Figure S4. UV−Vis absorption spectra of the cross-linked films of **V1498** and **V1499**.

FT-IR spectroscopy

FT-IR spectra were recorded by using a Perkin–Elmer Frontier spectrophotometer with a single reflectance horizontal ATR (Attenuated Total Reflectance) cell equipped with a diamond crystal. The data were recorded in the spectral range from 560 to 4000 cm^{-1} by accumulating 5 scans with a resolution of 4 cm^{-1}.

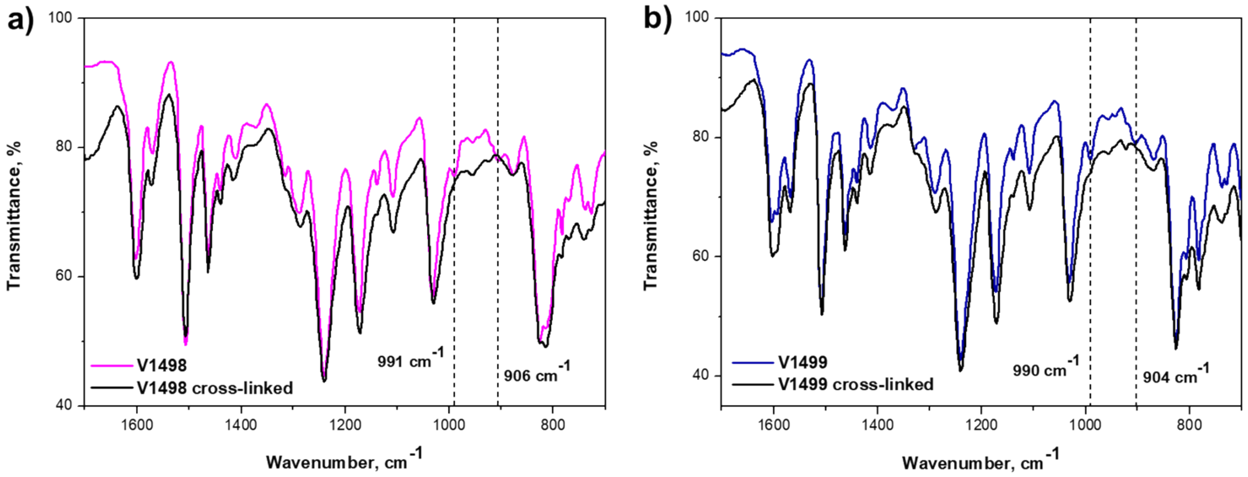

Figure S5. FT-IR spectra of neat and cross-linked **V1498** (a) and **V1499** (b).

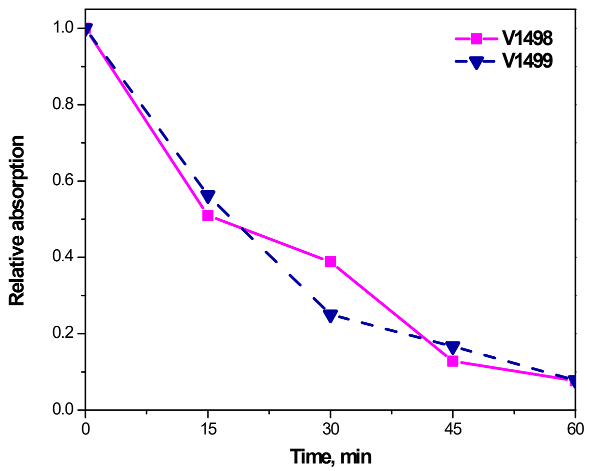

Figure S6. Cross-linking experiment of the **V1498** and **V1499** films. The maximum absorption of the solutions, prepared by dipping spin-coated HTM films into THF after heating for the respective times, relative to the absorption of the solution, prepared by dipping of the non-cross-linked film.

NMR spectra

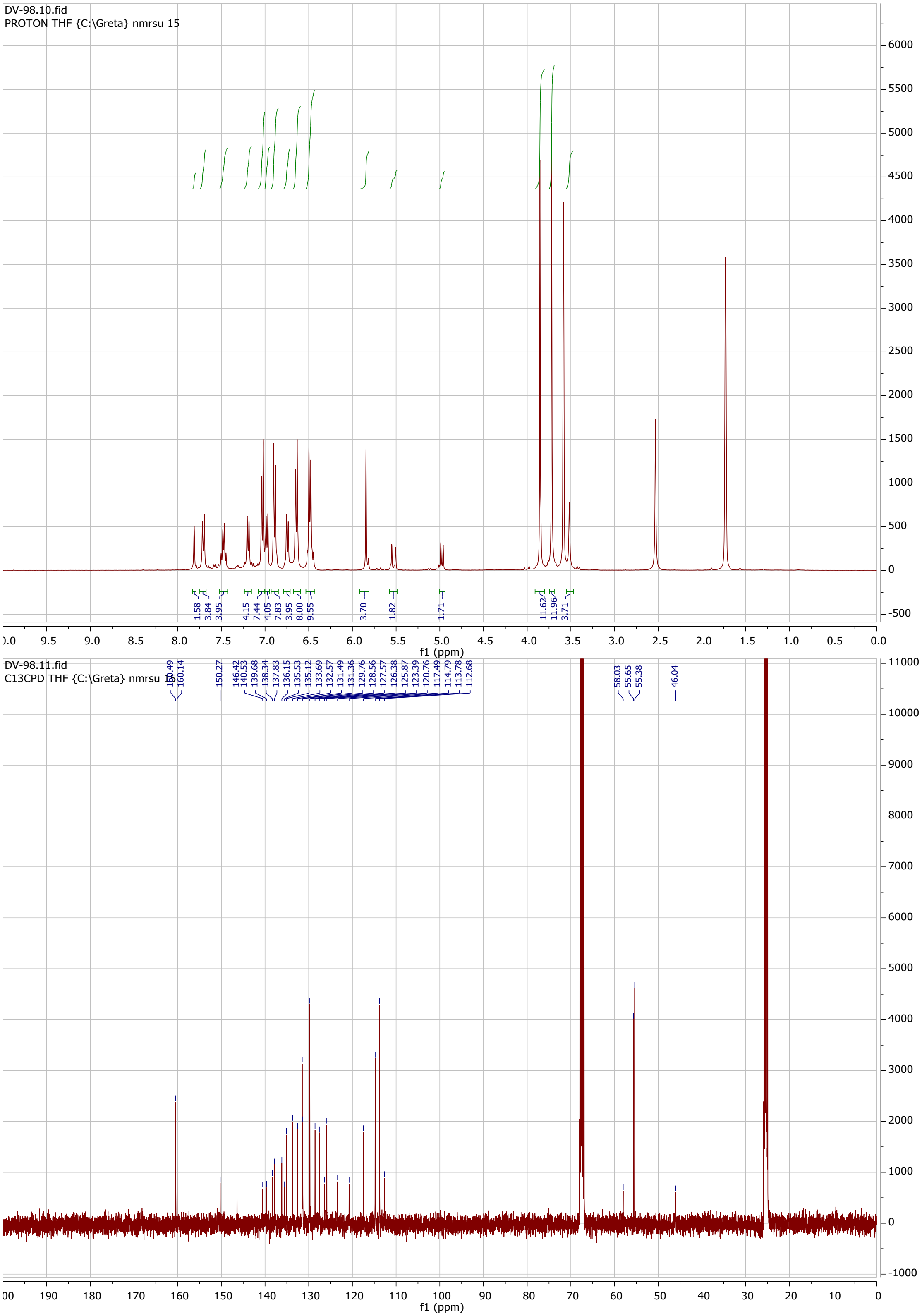

Figure S7. ^{1}H and ^{13}C NMR spectra of **V1498** in THF-d_8.

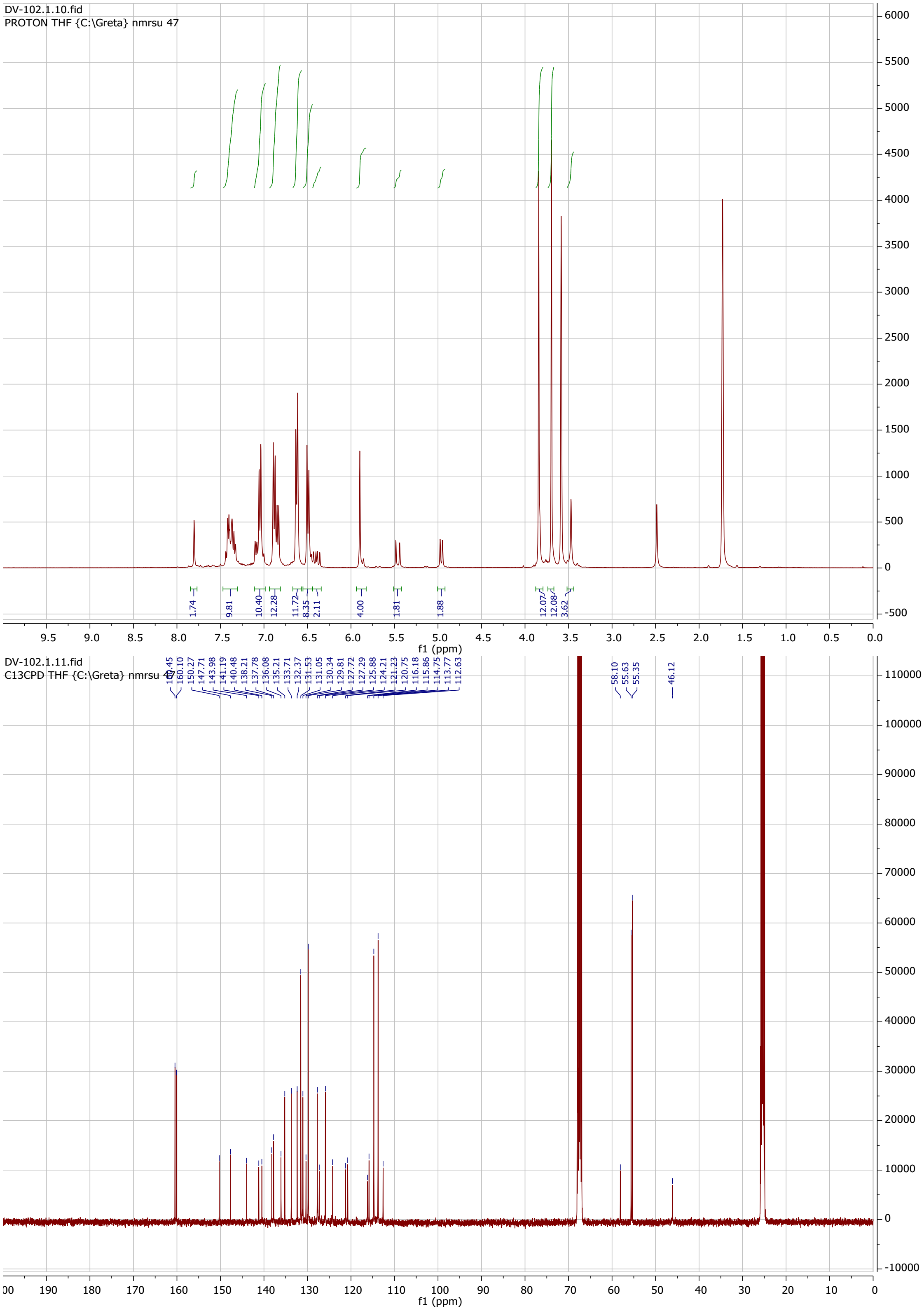

Figure S8. ^{1}H and ^{13}C NMR spectra of **V1499** in THF-d_8.

Synthesis cost

Table S1. Materials, quantities and cost for the synthesis of **V1498**.

Chemical	Weight reagent (g/g)	Weight solvent (g/g)	Weight workup (g/g)	Price of chemical (€/kg)	Cost of chemical (€/g product)	Total cost (€/g)
4-Bromoanisole	3.98			25.2	0.10	
Formic acid	20			3.87	0.08	
Sulfuric acid	4			2.63	0.01	
Methyl methoxyacetate	0.739			304	0.22	
Magnesium	0.518			104	0.05	
Diethyl ether		200		6.92	1.38	
Ethyl acetate			150	2.85	0.43	
**2,2-bis(4- methoxyphenyl)acetaldehyde**	29.237	200	150			2.28
2,7-Dibromofluorene	1.67			4490	7.50	
4-aminophenylboronic acid pinacol ester	2.25			2110	4.75	
Anhydrous tetrahydrofuran		67		213	14.27	
Anhydrous toluene		67		102	6.83	
Sodium hydroxide	3.08			38.08	0.12	
$PdCl_2(PPh_3)_2$	0.9			23400	21.06	
Celite			10	24.65	0.25	
Tetrahydrofuran			140	8.88	1.24	
Ethanol			80	6.08	0.49	
**4,4'-(9*H*-fluorene-2,7-diyl)dianiline (1)**	7.9	134	230			56.50
4,4'-(9*H*-fluorene-2,7-diyl)dianiline	0.45			56504.19	25.43	
2,2-bis(4- methoxyphenyl)acetaldehyde	1.73			2280	3.94	
10-Camphorsulfonic acid	0.45			260	0.12	
Toluene		18		2.46	0.04	
Ethanol			350	6.08	2.13	
Tetrahydrofuran			242	8.88	2.15	
n-Hexane			760	3.16	2.40	
Silicagel			50	64.8	3.24	
**4,4'-(9*H*-fluorene-2,7-diyl)bis(*N*,*N*-bis(2,2-bis(4-methoxyphenyl)vinyl)aniline) (2)**	2.63	18	1402			39.45
4,4'-(9*H*-fluorene-2,7-diyl)bis(*N*,*N*-bis(2,2-bis(4-methoxyphenyl)vinyl)aniline)	1.14			39451.12	44.97	
Benzyltriethylammonium chloride	0.018			159.8	0.00	
4-Vinylbenzylchloride	0.27			582.64	0.16	
Sodium hydroxide	0.18			38.08	0.01	
Dimethyl sulfoxide		45		47.27	2.13	
Ethyl acetate			200	2.85	0.57	
Na_2SO_4			50	6.08	0.30	
Tetrahydrofuran			282	8.88	2.50	
Ethanol			80	6.08	0.49	
n-Hexane			720	3.16	2.28	
Silicagel			50	64.8	3.24	
**V1498**	1.608	45	1382			56.65

Table S2. Materials, quantities and cost for the synthesis of **V1499**.

Chemical	Weight reagent (g/g)	Weight solvent (g/g)	Weight workup (g/g)	Price of chemical (€/kg)	Cost of chemical (€/g product)	Total cost (€/g)
4-Bromoanisole	3.98			25.2	0.10	
Formic acid	20			3.87	0.08	
Sulfuric acid	4			2.63	0.01	
Methyl methoxyacetate	0.739			304	0.22	
Magnesium	0.518			104	0.05	
Diethyl ether		200		6.92	1.38	
Ethyl acetate			150	2.85	0.43	
**2,2-bis(4- methoxyphenyl)acetaldehyde**	29.24	200	150			2.28
2,7-Dibromofluorene	1.43			4490	6.42	
3-aminophenylboronic acid pinacol ester	1.93			19600	37.83	
Anhydrous tetrahydrofuran		57		213	12.14	
Anhydrous toluene		57		102	5.81	
Sodium hydroxide	2.65			38.08	0.10	
$PdCl_2(PPh_3)_2$	0.77			23400	18.02	
Celite			10	24.65	0.25	
Tetrahydrofuran			120	8.88	1.07	
Ethanol			60	6.08	0.36	
**3,3'-(9*H*-fluorene-2,7-diyl)dianiline (3)**	6.78	114	190			82.00
3,3'-(9*H*-fluorene-2,7-diyl)dianiline	0.75			81999.51	61.50	
2,2-bis(4- methoxyphenyl)acetaldehyde	2.31			2280	5.27	
10-Camphorsulfonic acid	0.99			260	0.26	
Toluene		30		2.46	0.07	
Ethanol			500	6.08	3.04	
Tetrahydrofuran			282	8.88	2.50	
n-Hexane			720	3.16	2.28	
Silicagel			50	64.8	3.24	
**3,3'-(9*H*-fluorene-2,7-diyl)bis(*N*,*N*-bis(2,2-bis(4-methoxyphenyl)vinyl)aniline) (4)**	4.05	30	1552			78.16
3,3'-(9*H*-fluorene-2,7-diyl)bis(*N*,*N*-bis(2,2-bis(4-methoxyphenyl)vinyl)aniline)	1.14			78156.99	89.10	
Benzyltriethylammonium chloride	0.027			159.8	0.00	
4-Vinylbenzylchloride	0.33			582.64	0.19	
Sodium hydroxide	0.2			38.08	0.01	
Dimethyl sulfoxide		56		47.27	2.65	
Ethyl acetate			200	2.85	0.57	
Na_2SO_4			50	6.08	0.30	
Tetrahydrofuran			282	8.88	2.50	
Ethanol			80	6.08	0.49	
n-Hexane			720	3.16	2.28	
Silicagel			50	64.8	3.24	
**V1499**	1.697	56	1382			101.33

References

1 D. Vaitukaityte, C. Momblona, K. Rakstys, A. A. Sutanto, B. Ding, C. Igci, V. Jankauskas, A. Gruodis, T. Malinauskas, A. M. Asiri, P. J. Dyson, V. Getautis and M. K. Nazeeruddin, *Chemistry of Materials*, 2021, **33**, 6059–6067.

2 E. Miyamoto, Y. Yamaguchi and M. Yokoyama, *Electrophotography*, 1989, **28**, 364.

3 M. Cordona and L. Ley, *Top. Appl. Phys.*, 1978, **26**, 1.

4 E. Montrimas, V. Gaidelis and A. Pazera, *Lith. J. Phys.*, 1966, **6**, 569.

5 S. M. Vaezi-Nejad, *International Journal of Electronics*, 1987, **62**, 361–384.

6 A. Y. C. Chan and C. Juhasz, *International Journal of Electronics*, 1987, **62**, 625–632.

7 A. Al-Ashouri, M. Marčinskas, E. Kasparavičius, T. Malinauskas, A. Palmstrom, V. Getautis, S. Albrecht, M. D. McGehee and A. Magomedov, *ACS Energy Lett*, 2023, **8**, 898–900.
